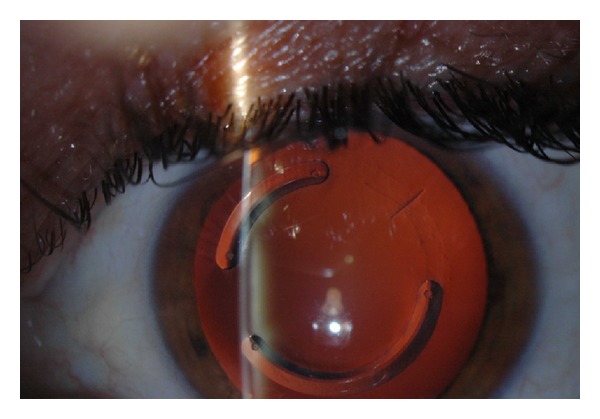# Random Implantation of Asymmetric Intracorneal Rings

**DOI:** 10.1155/2014/839359

**Published:** 2014-02-23

**Authors:** Cristina Peris-Martínez, Irene Gregori Gisbert

**Affiliations:** Fundación Oftalmológica Mediterráneo (FOM), Cornea and Refractive Surgery Unit, 46015 Valencia, Spain

## Abstract

Intracorneal ring employment for treating ectasia is widespread. Although the mechanism of action of intracorneal rings in the regularization of the corneal surface after its implantation is well known in most cases, there are still many doubts. We present a case of implanted intracorneal rings, where, despite the peculiar position of the rings, the patient gains lines of visual acuity and keratoconus remains stable.

## 1. Introduction

Keratoconus is the most common corneal ectasia in which central or paracentral thinning occurs without presenting signs of inflammation; it induces irregular corneal astigmatism and myopic shift [1].

The use of spectacles and contact lenses is sufficient to correct the refractive error in the initial stages. However, in more advanced cases, the therapeutic options may be different, ranging from intrastromal ring implantation, the use of cross-linking techniques, and lamellar keratoplasty to finally, in advanced cases, penetrating keratoplasty [2].

## 2. Clinical Case Report

A 48-year-old woman, with no relevant personal systemic history, was diagnosed with keratoconus in both eyes. Her ophthalmologic history presented penetrating keratoplasty of her right eye (RE) performed ten years previously due to advanced keratoconus and myopia magna of 12 D.

**Figure 1:**
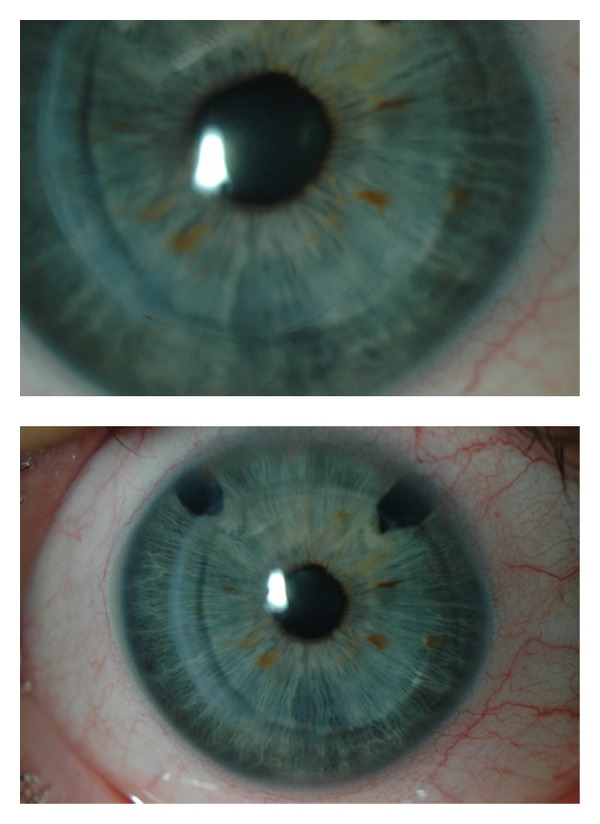
Right eye image of the patient by slit lamp, which shows penetrating keratoplasty and two upper iridectomies.

The patient's visual acuity (VA) was 0.2 with (−14.5, −3 × 100°) in her RE and 0.5 with (−10.75, −3.75 × 100°) in her left eye (LE). Ocular pressure was 16 mm Hg in her RE and 17 mm Hg in her LE. Slit lamp examination showed that the corneal button of her RE was clear, with no signs of rejection or vascularization at the edges (Figure 1). Her LE clearly presented an advanced keratoconus with signs of Vogt's striae, an iron line forming a Fleischer ring, and the presence of prominent intrastromal corneal nerves.

The funduscopic examination revealed the existence of advanced myopic retinochoroid it is with paripapillary and retinal atrophy areas in both eyes.

Even though the stage of the keratoconus was so advanced in her LE, two intrastromal rings were implanted by IntraLase femtosecond laser. We used the femtosecond laser to make the channel and the corneal incision where the intracorneal rings were inserted. To introduce the corneal rings into the unique corneal channel, specific forceps and Sinskey hook were employed. Two symmetrical rings were placed, one upper and one lower, with a triangular-shaped cross-section like the Keraring model, 300 microns thick, 160° arch length, axis at 24°, and at a depth of 371 microns. There were no surgical complications.

**Figure 2:**
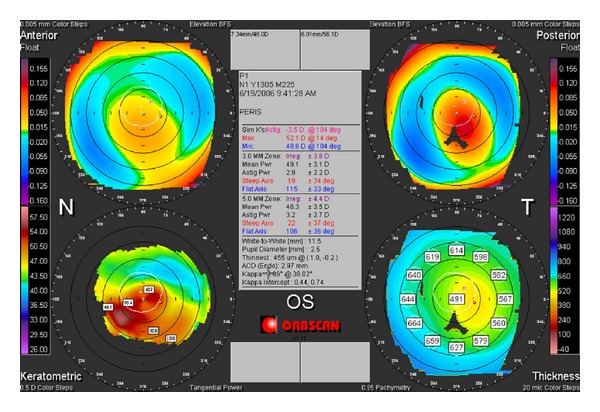
Corneal topographic image of the left eye of the patient after implantation of the corneal rings.

After implanting the rings, her corrected VA was 0.6 with (−12, −2 × 90°) in her LE and corneal topography showed a slight improvement in ectasia with a lower apex, mean pachymetry of 455 microns; the maximum keratometry was 52.1 mm and the minimum was 48.6 mm, and astigmatism was −3 diopters (D) (Figure 2).

**Figure 3:**
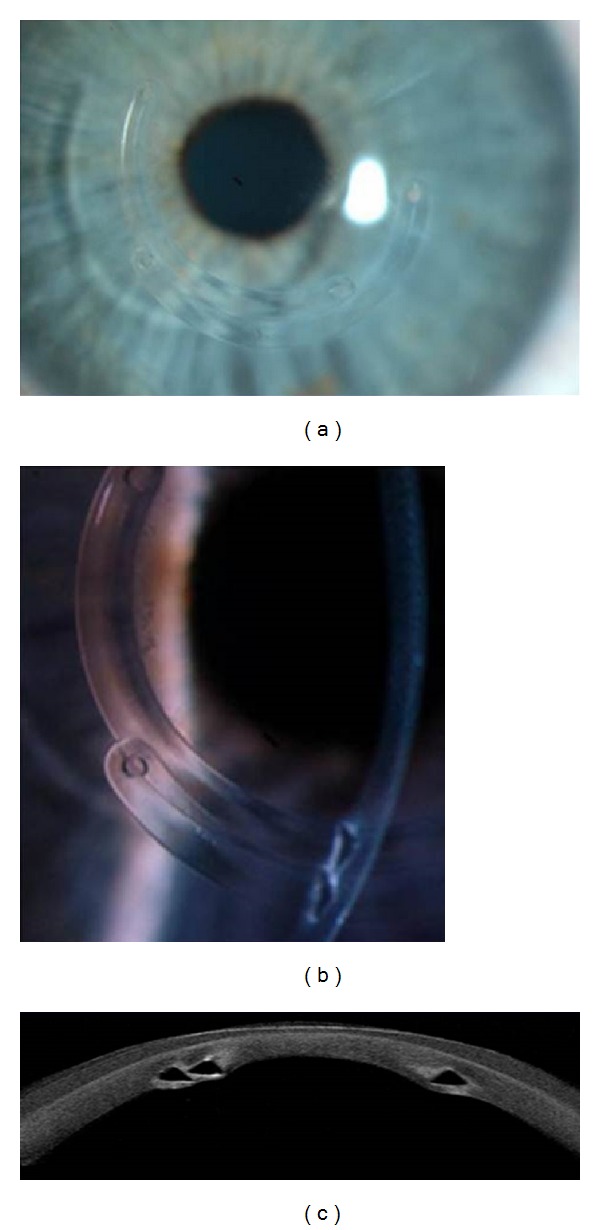
(a) Superior-left. Image of the patient's left eye by slit lamp, which shows the two implanted intracorneal rings, inferiorly embraced. The habit of scratching the patient probably mobilized within the channel rings. Three years after surgery the patient remains stable. (b) Superior-right. The same eye image where you can see the triangular profile of both rings implanted. (c) Inferior. By performing the anterior segment OCT-Visante, we can find the exact position of the rings. In this case the rings are located one above the other. Currently the patient carries contact lens to correct residual refractive error.

Two years after ring surgery, in a routine check-up, the patient presented a corrected VA of 0.7 with (−12, −1 × 90°), so she had gained one line of vision. Slit lamp examination revealed the displacement of the upper ring in the lower area next to the other ring (Figures 3(a) and 3(b)), which was confirmed by coherence tomography of the anterior pole (Visante) (Figure 3(c)). To clarify the usual normal position of intracorneal rings, we can see the photograph with normally positioned intracorneal rings in another patient (Figure 4). Topography did not show a progression in corneal thinning but presented pachymetry of the apex of 483 microns, maximum keratometry of 51.5 mm and 47.8 mm, and astigmatism of −2 D.

Since the patient's ectasia has not increased and her VA has improved, she will simply be observed and no move will be made to reposition the ring in the upper area.

## 3. Discussion

Keratoconus is a corneal ectasia that affects the structure and transparency of the cornea, causing significant visual problems. This progressive disease is usually bilateral and is associated with irregular astigmatism which, on occasions, is difficult to compensate with lenses thus; other, techniques have to be used to stop the progression of corneal thinning. Screening for incipient keratoconus in people who are going to have refractive surgery is essential as corneal ectasia associated with LASIK has been described. Therefore, determination of corneal indexes with topography is vital for all patients to discard the presence of keratoconus suspects.

Currently, there are different treatment options for corneal ectasia before resorting to penetrating keratoplasty, which can lead to transplant rejection, a significant loss of endothelial cells (especially if life expectancy is long), irregular astigmatism, secondary effects of corticoids (e.g., secondary glaucoma and cataracts), and recurrence of keratoconus.

Intrastromal rings are a new therapeutic option. Their purpose is to flatten and regularize the surface of the cornea to improve or stabilize irregular astigmatism and to slow down the progression of keratoconus, apart from providing better visual acuity.

There are different types of rings, such as the Keraring-Ferrara and the INTACS. The former are polymethylmethacrylate (PMMA) rings with a triangular-shaped cross-section and they are implanted at a diameter of 5-6 mm. The INTACS are also made of PMMA, but the cross-section is hexagonal-elliptical and they are implanted forming a diameter of 6-7 mm. The Kerarings are manufactured with a thickness of 0.15 and 0.30 mm and different length arches (90°, 150°, 160°, and 210°) [2]. In our case, the Kerarings were used at 5 mm diameter (0.6 mm triangle base).

Personalized implantation of the rings can be symmetrical or asymmetrical, with one ring or two, depending on the patient and taking a nomogram as the basis.

Until a few years ago, the rings were implanted manually with greater complications due to the learning curve, such as epithelial defects, anterior and posterior corneal perforations, infectious keratitis, stromal corneal edema around the incision, extension of the incision towards the visual axis, and displacement of the rings. With the appearance of new technologies, both the creation of the intrastromal channel and the insertion incision can be made by femtosecond laser (IntraLase, [3]). In this way surgery duration is minimized and the risk of inflammation and infection is reduced. Exceptional intraoperative complications can include the formation of an incomplete channel, in which case it is carried out manually with a mechanical separator, the risks being endothelial perforation or a false channel. Postoperative complications can include the migration and shift of the segments, corneal melting, or infection after the implantation of the rings like, for instance, the formation of a corneal abscess at the point of incision. The most usual of the above-mentioned complications is the migration of rings postoperatively [4].

In this clinical case, the upper ring has migrated and shifted and now mounts the lower one partially. But paradoxically, this has not proved to be a complication, but rather the patient shows an improvement in her visual acuity and, to date, it does not appear to affect the corneal thinning process (three years after implantation).

One of the aspects of maximum debate is the point at which the incision should be made prior to intracorneal ring implantation, so that the rings are most effective. The incision can be made at either the most curved astigmatic axis or the comatic axis [5], as this is the high order aberration that is most usually found to be affected in some keratoconuses. It is usually inserted in the most curved astigmatic axis, but when there is a great difference between the comatic axis and the astigmatic axis, implantation in the comatic axis is recommended to achieve a greater effect. In this case, we made an incision in the most curved astigmatic axis. We did not have an aberrometric map for this patient prior to surgery, so we do not know whether the patient had a high preoperative coma. In view of the improved visual acuity the patient achieved after the ring shift (bear in mind that IntraLase laser makes a single channel when implanting the rings, femtosecond unlike implantation performed manually), we presume that the ring was implanted asymmetrically and that it compensates the high preoperative coma.

The patient did not suffer any prior traumatism, so we believe the ring may have shifted in the interior of the channel because she rubbed her eyes, as these patients often have this habit and sometimes do it unconsciously.

To conclude, intrastromal rings are an alternative to keratoplasty in primary corneal ectasias or after refractive surgery. The technique with femtosecond laser is minimally invasive, the most common complication reported by studies being the migration of the segments, which on occasions, such as in this case, can mean an improvement for the patient when they do not migrate outwards, but rather within the channel.

## Figures and Tables

**Figure 4:**